# Folate and Its Impact on Cancer Risk

**DOI:** 10.1007/s13668-018-0237-y

**Published:** 2018-08-11

**Authors:** Renee Pieroth, Stephanie Paver, Sharon Day, Carolyn Lammersfeld

**Affiliations:** 1 Department of Nutrition, Cancer Treatment Centers of America, 1331 East Wyoming Ave, Philadelphia, PA 19124 USA; 2 RD, LLC 10645 N. Tatum Blvd., Suite 200, Mailbox 122, Phoenix, Arizona, 85028 USA; 3 Department of Nutrition, Cancer Treatment Centers of America, 14200 W. Celebrate Life Way, Goodyear, Arizona, 85338 USA; 4 Department of Medicine and Science, Cancer Treatment Centers of America, 2610 Sheridan Road, Zion, IL 60099 USA

**Keywords:** Folate, Cancer risk, Folic acid, Folate deficiency, Folate supplementation, MTHFR, SHMT, Serum folate

## Abstract

**Purpose of Review:**

Research has evaluated the potential impact of folate on cancer risk with conflicting findings. Studies have demonstrated increased risk, no effect, and decreased risk. This review summarizes findings of mixed results between folate intake, serum levels, gene polymorphisms, and cancer risk based on meta-analyses from the past five years.

**Recent Finding:**

Low or deficient folate status is associated with increased risk of many cancers. Folic acid supplementation and higher serum levels are associated with increased risk of prostate cancer. Gene polymorphisms may impact risk in certain ethnic groups.

**Summary:**

Folate has been studied extensively due to its role in methylation and nucleotide synthesis. Further prospective studies are needed to clarify optimal levels for nutrient remediation and risk reduction in those at risk, as well as elucidate the association between high intake, high serum levels, and prostate cancer risk. Future considerations for cancer risk may include gene interactions with nutrients and environmental factors.

## Introduction

Folate is an essential water-soluble B vitamin found in foods, including dark-green leafy vegetables and legumes. Folic acid is the synthetic form of the vitamin present in supplements and fortified foods, namely grains and cereals. Dietary folate exists in a reduced state with polyglutamate side chains requiring oxidation and hydrolysis for absorption, whereas folic acid exists as the oxidized pteroylmonoglutamate form making it readily bioavailable [1]. Dietary folate bioavailability ranges from 10 to 98% and is influenced by intestinal pH, enzymatic activity, presence of alcohol and other inhibitors, malabsorption disorders, and the food matrix [1]. To account for the differences in absorption between folate and folic acid, folate equivalents are used. The recommended dietary allowance for adults in the USA is set at 400 micrograms (mcg) of dietary folate equivalents (DFE). One microgram of DFE is equivalent to 1 mcg dietary folate, 0.6 mcg folic acid consumed with food, or 0.5 mcg supplemental folic acid on an empty stomach. The upper limit for folate is 1,000 mcg/day, which is one fifth of the minimum amount known to mask a B_12_ deficiency [2, 3]. About 5% of the US population gets more than the upper limit, usually due to dietary supplements [4]. There are several ways to assess folate status. Serum levels, are thought to reflect recent intake. Deficiency is categorized as less than 7 nmol/L to less than 10 nmol/L. Red blood cell (RBC) folate represents folate status over months with levels less than 315 to 363 nmol/L suggesting deficiency [1, 3, 5]. Elevated urinary formiminoglutamate (FIGLU) excretion and deoxyuridine suppression tests are additional methods for assessing folate status. Also, elevated homocysteine may be a functional measure of folate status [1].

Due to its role in one-carbon metabolism, folate has been studied extensively as a possible mechanism for cancer development. Folate as 5-Methyltetrahydrofolate (5-MTHF) and cobalamin are required for the conversion of homocysteine to methionine in the methionine pathway. Methionine is converted to *S*-adenosylmethionine (SAM). SAM is a chief methyl contributor to many reactions in the body, including DNA and RNA methylation [6]. Inadequate production of SAM may lead to decreased methylation of CpG islands in DNA affecting gene transcription, altering expression of tumor suppressor genes and proto-oncogenes [6, 7]. Furthermore, folate deficiency can impair conversion of deoxyuridine monophosphate (dUMP) to deoxythymidine monophosphate (dTMP), the nucleic acid necessary for DNA synthesis and repair [6, 7]. The misincorporation of uracil for thymidine can eventually lead to unstable DNA, DNA strand breaks, and faulty DNA repair [6, 7].

Excess folate intake also poses a concern. Mandatory fortification of wheat flour and enriched cereal grain products aimed at reducing the incidence of neural tube defects has raised questions regarding growth of nascent cancers [3]. Mandatory fortification has been in effect in the USA since 1998, and at least 87 countries have regulations in different stages of implementation [8]. In the USA, a 140 mcg/100 g product is added to fortified foods and in some countries more is added; for example, Chile adds 220 mcg/100 g product [3]. In addition, 35% of US adults may take a supplement containing 400 mcg folic acid, and in some countries, the frequency of people supplementing is even higher. Average total folate intake of 813 mcg/day for men and 724 mcg/day for women has been reported in the USA, with only 15–17% of adults not meeting the daily recommended amount [4].

Folic acid, found in fortified foods and supplements, is converted to tetrahydrofolate in the liver by dihydrofolate reductase (DHFR). It has been reported that folic acid in excess of 400 mcg may saturate the DHFR enzyme, resulting in unreduced folic acid, which has been hypothesized as a potential mechanism for carcinogenesis [3, 9•].

Just as overt folate deficiency and excess intake may interfere with cell replication and survival, reduced enzyme efficiency can also interfere with nutrient metabolism and influence disease risk. Methylenetetrahydrofolate reductase (MTHFR) is a key flavoenzyme that catalyzes the reduction of 5,10-methylenetetrahydrofolate to 5-methyltetrahydrofolate (5-MTHF). It is encoded by the gene MTHFR. There are two well-described MTHFR gene polymorphisms: C677T and A1298C. The C677T variant may occur in close to 20–40% of the population [12, 53]. Both variants are associated with reduced enzyme activity [11]. Enzyme efficiency is reduced by up to 45% for the 677CT variant and by up to 70% for 677TT [10]. The variated form of the enzyme has less affinity to its cofactor, flavin adenine dinucleotide. Cytosolic serine hydroxy methyl transferase (SHMT1) is another key enzyme in folate metabolism. It converts serine and tetrahydrofolate to glycine and 5,10-methylenetetrahydrofolate, respectively. 5,10-methylenetetrahydrofolate is a substrate for purine and pyrimidine synthesis. The combination of folate deficiency and polymorphisms may result in DNA hypo- [12] or hypermethylation [13].

The relationship between folate and cancer risk remains uncertain, as studies have demonstrated positive, negative, and neutral associations. Also of note, antifolates are used in cancer treatment, however that is outside of the scope of this review. Therefore, the purpose of this paper is to summarize current knowledge of folate’s impact on cancer risk and identify opportunities for future work in this area based on a review of meta-analyses from the past 5 years. Many factors contribute to the findings, including the type and dose of the vitamin (dietary, supplemental, and total), serum levels and method of evaluating, cancer type and presence of gene polymorphisms.

## Methods

This review was conducted to evaluate the impact of folate on cancer risk. PubMed/MEDLINE and the Cochrane database were searched by combining the key words “folate” or “folic acid” and “cancer risk.” Meta-analyses published in the last 5 years in human adults and in English were included. Titles and abstracts were reviewed to select articles related to cancer risk in adults. Articles were excluded if they were related to cancer treatment, childhood cancer risk, or other disease states. Additional articles were obtained from references of relevant papers. A summary of the review articles selected is shown in Tables 1 and 2.

**Table 1:** Literature search of meta-analyses investigating folate and cancer risk in humans

First author, reference	Number of cases/controls (cohort)	Cancer type	Measurements	Results
Chen P [14]	Folate intake and breast cancer risk	Breast	*Prospective studies* Dietary folate intake	NS
	16 prospective: 744,068 participants/26,205 breast cancer		Higher total folate intake	NS
				U-shaped
	26 case-control: 16,826 cases/21,820 controls.		Dose-response	S: reduced risk with dietary folate intake 153–400 mcg compared with those of < 153 mcg. NS: reduced risk with intake > 400 mcg compared to < 153 mcg
	Serum folate level and breast cancer risk 8 studies: 5,924 participants			
			*Case-control studies* Dietary folate intake	S: reduced risk dietary folate intake highest category compared to lowest category NS when adjusted for publication bias
			Higher total folate intake	NS
			Dose-response	S: increased intake of 100 mcg dietary folate per day showed reduced breast cancer risk
Zhang YF [15]	14 prospective observational studies	Breast	Folate intake	NS
	677,858 participants		Dose response	NS with 100 mcg per day; heterogeneity S: J-shape correlation between folate intake and breast cancer risk. Dietary folate intake 200–320 mcg was associated with lower breast cancer risk; risk increased with daily folate intake > 400 mcg
			Subgroup analyses: country, study design, sample size, effect estimate, follow-up duration, adjusted alcohol intake	S: higher folate intake associated with reduced risk compared to lowest category if daily alcohol intake > 10 g S: folate intake associated with increased risk when nested case-control studies included; 100 mcg per day increment in folate intake was associated with increased risk.
Liu [16]	15 prospective cohort studies, 1 nested case-control study.	Breast	Dietary folate	NS
	1,854,013 participants and 24,620 breast cancer cases.		Dose-response	NS: 220 mcg per day increment in dietary folate intake was not associated with breast cancer risk
			Menstrual status, hormonal status, and consumption of alcohol, methionine and vitamin B12.	NS
Tio [52]	36 studies.	Breast	Dietary folate	NS: once adjusted
	608,265 sample size, 34,602 cases		Total folate intake	NS
			Menopausal status	NS once adjusted
			Hormone status	NS
			Study design	S: retrospective study design showed decreased risk of postmenopausal breast cancer
Fan C [18]	4,090/10,902 9 case-control studies: 7 dietary folate with FFQ; 2 studies serum folate concentration; 4 studies dose-response analysis	Head and neck	Folate intake	S: decreased risk comparing highest to lowest folate intake
			Dose-response	S: every 100 mcg/day increase in folate intake associated with 4.3% decreased risk
Galeone C [19]	5,127/13,249 Pooled analysis of individual-level data from 10 case-control studies	Oral cavity and pharyngeal (OPC)	Total folate intake	S: Reduced risk for highest compared lowest quintile total folate intake and overall OPC risk S: decreased risk for oral cavity
			Alcohol	S: increased risk for heavy drinkers with low folate intake compared to low alcohol intake with higher folate intake
			Smoking	S: increased risk for smoking
Zhao Y [20]	19 studies	Esophageal	Dietary folate intake	S: decreased risk with highest folate intake compared to lowest intake
	14 case-control 1 cohort dietary folate intake			
	6 case-control 1 cohort for dose-response		Dose-response	S: 100 mcg/day increment in dietary folate intake reduced risk by 12%
Tio M [21]	9 case-control 2574/9254 for any histological type of esophageal cancer.	Esophageal, gastric, pancreatic	Dietary folate	S: dietary folate associated with decreased risk of esophageal and pancreatic cancer NS: gastric cancer
	8 studies (5 prospective, 3 retrospective) 2209/295,526 for pancreatic cancer.			
	16 for gastric (3 prospective, 13 retrospective) 4414/209,689			
Lin HL [22]	13 studies with 14 estimates (7 cohort, 6 case-control); 10 for dietary folate; 5 supplemental; 4 total, 3 serum	Pancreatic	Dietary folate intake	S: decreased pancreatic cancer risk with highest dietary folate intake compared to lowest intake
	*N* = 3467		Supplemental	NS
	2 case-control, 5 cohort for dose-response analysis		Dose-response	S: decreased risk for pancreatic cancer with 100 mcg/day increase intake
Zhang YF [23]	9 cohort studies with *n* = 566,921	Lung	Dietary folate intake	NS
			Dose-response	NS per 100 mcg/day increase
			Dietary folate intake	S: low intake reduced risk of lung cancer in women
			Dietary folate intake	S: high intake reduced risk in men
Wang R [24]	10 with *n* = 202,517	Prostate	Dietary folate intake	NS
	5 cohort dietary folate		Dose-response	NS: dose response of 100 mcg/day
Tio M [17]	19 studies	Prostate	Dietary folate	NS: marginally decreased risk
	11 (5 cohort, 6 case-control) for dietary folate intake 15,336/146782		Total folate	NS: no association
	5 (2 cohort, 3 case-control) Total folate intake 7114/93781			
He H [25]	13 studies (7 cohort, 6 case-control) 6280 cases	Bladder	Total folate intake	S: decreased risk of bladder cancer associated with highest compared to lowest folate intake.
			Dietary folate	S: decreased risk of bladder cancer with dietary folate
			Study design	S: inverse association between folate intake and bladder cancer in case-control studies; NS in cohort studies
Li C [26]	Folate intake: 8 studies highest quantile vs lowest folate intake and risk	Ovarian	Dietary folate	S for high dietary folate and reduced risk when 1 study removed due to heterogeneity
	1158 cases out of 217,309 4 cohort studies folate intake 4519/6031 case-control folate intake		Total folate	NS
Du L [27]	9 case-control and 5 cohort studies	Endometrial	Total folate	NS
			Dose-response	S: in the highest category, 5% increased risk per 100 mcg/day
			Study design	S: decrease risk of total folate intake in case-control studies; NS in cohort studies
			Country	NS in North American studies; S: decrease risk of total folate intake in studies outside of North America (China, Mexico, Switzerland)

S = statistically significant (*P* < 0.05)

NS = not statistically significant (*P* > 0.05)

**Table 2:** Literature search of meta-analyses investigating serum folate levels, folate supplementation, gene polymorphisms, and cancer risk

First author, reference	Number of cases/controls (cohort)	Cancer type	Measurements	Results
Chen P [14]	Serum folate level and breast cancer risk	Breast	Serum folate	NS: prospective studies;
				NS: case-control studies
	8 studies: 5,924 participants		Folic acid supplemental	NS
	Folate intake and breast cancer risk 16 prospective: 744,068 participants/26,205 breast cancer 26 case-control: 16,826 cases/21,820 controls.		Stratification: study quality, menopause status, estrogen receptor status, alcohol intake, race/ethnicity	NS: prospective studies had heterogeneity for the summary estimate between the stratification; S: Case-control studies had reduced risk with higher dietary folate in Europe, Australia or Asia but not in the USA S: hospital-based studies suggested highest category of dietary folate intake showed reduced breast cancer risk; no association with population-based studies S: higher quality studies suggested higher folate intake may reduce breast cancer risk NS menopausal status S: increase dietary folate intake reduced breast cancer risk for women with higher alcohol intake, but no association for those with lower alcohol intake
Tio [52]	36 studies.	Breast	Serum folate	NS
	608,265 sample size, 34,602 cases			
Zhao Y [20]	19 studies 4 for serum folate	Esophageal	Serum folate	S: decreased risk for highest vs lowest serum folate
Tio M [21]	9 case-control 2,574/9,254 for any histological type of esophageal cancer.	Esophageal, gastric, pancreatic	Serum folate	NS: Serum levels and pancreatic cancer
	8 studies (5 prospective, 3 retrospective) 2,209/295,526 for pancreatic cancer.			
	16 for gastric (3 prospective, 13 retrospective) 4,414/209,689			
Lin HL [22]	13 studies with 14 estimates (7 cohort, 6 case-control); 5 supplemental N = 3,467	Pancreatic	Serum folate	NS: decreased risk
			Folic acid supplementation	S: decreased risk for 100 mcg/day increment increase intake
Wang R [24]	10 studies. n = 202,517	Prostate	Serum folate	S: high levels serum folate levels were associated with increased risk
	5 nested case-control			
			Dose-response	S: 5 nmol/L increment associated with increased risk
Tio M [17]	7 (6 nested case-control, 1 case-control) 6,122/10,232	Prostate	Serum folate	S: high levels associated with increased risk
Zhou X [28]	6 case-control 873/1,510	Cervical	Serum folate	S: deficient serum folate levels and increased risk; S: increased risk if folate level ≤ 6.4 ng/ml; NS: If deficiency level ≥ 6.4 ng/ml
			Sample size	NS: studies with *n* ≥ 500; S: if sample size < 500
			Country	NS: American studies; S: Asian studies
Zhang D [11]	16 studies 5,657/6,557 MTHFR C677T, A1298C, G1793A	Overall cancer risk	Serum homocysteine	S: increased risk with high homocysteine
			Serum folate	S: increased risk with folate deficiency
S.Chuang [29]	8 publications (10 cohorts, representing 3,477 cases/7,039 controls)	CRC	Serum folate	NS: higher levels compared to lowest and risk S: inverse association between circulating folate and CRC risk in studies that used the radioimmunoassay.
D.Kennedy [30]	67 studies met criteria; 62 case controlled, 2 cohort and 3 older versions retained for folate intake data	CRC	MTHFR polymorphisms	S: reduced risk of CRC with high total folate intake with 677TT genotype, 677CC NS: A1298C
He H [25]	13 studies (7 cohort, 6 case-control) 6,280 cases	Bladder	Folic acid supplementation	NS
Li C [26]	Folate intake: 8 studies highest quantile vs lowest folate intake and risk	Ovarian	Folic acid (fortified foods and supplements)	NS
			MTHFR polymorphisms	NS
	1,158 cases out of 217,309 four cohort studies folate intake 4,519/6,031 case control folate intake 5,617/9,808 polymorphisms			
Mao B [31]	5 studies (7 cohorts) 2441/133,995	Renal	Folic acid supplementation	S: decreased risk with increase of 100 mcg/day FA supplementation
	3 case-control; 2 cohort; 1 nested case control; 1 case cohort			
Qin X [32•]	15 RCTs: 13 with total cancer incidence outcome and folic acid supplementation	Total incidence (13 trials, *n* = 49,406) Colorectal (7 trials, *n* = 33,824) Other GI (2 trials, n = 20,228) Prostate (5 trials, *n* = 27,065) Other GU (2 trials, n = 20,228) Lung (5 trials, *n* = 31,864) Breast (4 trials, *n* = 19,800) Heme (3 trials, *n* = 25,670) Total Cancer Mortality (6 trials, *n* = 31,930) Melanoma (3 trials, *n* = 19,128)	Folic acid supplementation	NS total cancer; colorectal; other GI; prostate; other GU; lung; breast; heme; total cancer mortality S: folic acid supplementation decreased risk of melanoma
			Lipid-lowering drugs	S: elevated risk with lipid-lowering drugs
			Hypertension	NS
Vollsett SE [33•]	13 trials *N* = 49,621	Overall cancer incidence from cardiovascular or colorectal adenoma prevention studies comparing folic acid to placebo over 5–6 years	Folic acid supplementation	NS: median dose of 2.0 mg folic acid
Wien TN [34•]	19 studies (12 RCT)	Cancer risk associated with	Folic acid supplementation	S: increased risk in prostate cancer
	10 RCT for overall incidence	folic acid supplementation		S: marginal increased risk in cancer incidence with FA
	6 RCT prostate			S: increased incidence in prostate cancer with FA S: marginal increased risk in cancer mortality with FA S: increased risk if doses between 0.4 and 1 mg, but not if > 1 mg
			Gender	S: increased risk in studies with > 70% males
			Current smokers	S: increased risk if > 30% smokers
			Follow-up < or > 60 months	S: increased risk if > 60 months of follow-up
T.Qin [35]	8 RCT	CRC	Folic acid supplementation	NS
R.Heine-Broring [36]	24–4 studies specific to folic acid (supplements and fortification) Systematic review and meta-analysis of prospective cohort studies	CRC	Folic acid supplementation	S: inverse association for highest vs lowest folic acid from supplements and colorectal cancer risk
			Dose-response	NS: no association for increase of 100 mcg/day of folic acid from supplements and CRC
Y.Liu [37]	47 articles met inclusion criteria (19 studies relating to folate)	CRC	Folic acid supplementation	S: reduced risk with highest compared to lowest intake
Li C [26]	MTHFR polymorphism: 10 studies with 12 subgroup studies: 5,617 cases/9808 controls	Ovarian	MTHFR polymorphisms	NS
Zhang D [11]	16 studies 5,657/6,557 MTHFR C677T, A1298C, G1793A	Overall cancer risk	MTHFR C677T, A1298C, G1793A	S: increased risk of C677T homogeneity and overall risk of cancer
Tang M [38]	134 case-control studies	MTHFR C677T and cancer risk	T vs C	S: increased risk
	46,207 cases and 69,160 controls		TC vs. CC	NS
			TT vs CC	S: increased risk
			TC + TT vs CC	S: increased risk
			TT vs. TC + CC	NS
			Race/ethnicity	S: increased cancer risks were indicated among Asians in all genetic models except for heterozygote model
			Cancer type	S: increased risks of esophageal and stomach cancer were observed across models
Rai [7]	14 case-control studies 9,468 cases, 9,078 controls	MTHFR C677T, Lung Asian population, China, Japan, Taiwan	T vs C	S: increased risk
			CT vs CC	NS
			TT vs CC	S: increased risk
			TT + CT vs CC	S: increased risk
			TT vs CT + CC	S: increased risk
Rai [12]	36 case-control studies 8,040 cases, 10,008 controls	MTHFR C677T, Breast Asian population, Turkey, China, Korea, Taiwan, E.As, India, Singapore, Japan, Arab, Iran, Syria, Pakistan	T vs C	S: increased risk
			CT vs CC	NS
			TT vs CC	S: increased risk
			TT + CT vs CC	S: increased risk
			TT vs CT + CC	S: increased risk
Kaya [39]	6 case-control studies 707 cases, 880 controls	MTHFR C677T, Breast Turkish populations	T vs C	S: increased risk
			TT vs. CC	S: increased risk
			TT + CT vs. CC	NS
			TT vs. CT + CC	S: increased risk
Yi [40]	12 case-control studies for C677T	Cervical	MTHFR C677T	NS
	2,332 cases and 3,000 controls 5 studies for A1298C polymorphisms 677 cases and 1,191 controls	Caucasian, Asian, and mixed descent. Studies had been carried out in China, Korea, India, Greece, Germany, The Netherlands, and Poland.	MTHFR A1298C	S: increased cervical cancer risk was found in 3 models: allele contrast, heterozygote, and dominant
			Race/ethnicity	S: increased cervical cancer risk among Asian descent
Li [26]	5 population-based case-control studies, 5 hospital/clinic-based case-control studies, 1 nested case-control 5,617 cases, 9,808 controls	MTHFR C677T, ovarian	Heterozygote, homozygote, dominant, and recessive.	NS
			Race/ethnicity	Asian ethnicity was associated with increased ovarian cancer risk for dominant, recessive and homozygous models
Lu [41]	10 case-control studies 1,786 cases, 2,076 controls	MTHFR C677T, Glioma	Allele contrast, heterozygote, homozygote, dominant, recessive.	NS
Xu [42]	8 case-control studies 3,059 cases, 3,324 controls	MTHFR C677T, Primary brain	T vs. C	NS
			TC vs. CC	S
			TT vs. CC	NS
			TT + TC vs. CC	S
			TT vs. CC + TC	NS
			Race/Ethnicity	NS: Caucasians; S: increased risk between the MTHFR C677T variant and the risk for brain tumors across all 5 models in Asians
			Tumor type	S: increased risk of meningioma associated with TC carriers compared with the CC carriers
Liang [43]	7 case-control studies 2,030 cases, 3,096 controls	MTHFR A1298C, Liver Caucasians, Asians	CC vs AA	S: decreased risk
			CC vs AA + AC	S: decreased risk
			Race/ethnicity	S: homozygote genotype CC of MTHFR rs1801131 polymorphism was associated with decreased risk of liver cancer in Asians
Qi [44]	7 case-control studies for C677T 5 case-control studies for A1298C Chinese population	Hepatocellular	MTHFR C677T	S: MTHFR C677T polymorphism was significantly associated with susceptibility to HCC in Chinese population
			MTHFR A1298C	S: MTHFR A1298C polymorphism was associated with decreased risk of HCC in Chinese population
Guo [45]	23 studies 11,348 cases, 12,676 controls	Prostate	MTHFR C677T	NS
			Ethnicity	S: decreased risk seen for Asians across all genetic models
			Study design	S: decreased risk in hospital-based studies for the homozygote and recessive models; in aggressive prostate cancer for the homozygote model
Jia [46]	7 case-control studies 1,318 cases, 1,817 controls	MTHFR C677T, Oral	CT vs. CC	NS
			Race/ethnicity	S: decreased risk associated with Asians CT vs CC
			Hospital-based studies	S: decreased risk in hospital-based studies TT vs. CC; CT vs. CC.
			Alcohol	S: increased risk heavy vs. non-heavy drinkers T vs. C; TT vs. CC; TT vs. CC; TT vs. CT + CC
Dong [47]	8 case-control studies 1,114 cases, 3,227 controls	Myeloid leukemia	MTHFR A1298C	NS
			Race/ethnicity	S: increased risk for Asians CC vs. AC + AA
Yang [48]	4 case-control studies 360 cases, 900 controls	Thyroid	MTHFR C677T	S: increased risk T vs. C; TT vs. CC
	Included populations among Iran, India, Turkey, and Saudi Arabia			
He [49]	25 studies for MTHFR C677T 7,448 cases, 11,146 controls 19 studies for the A1298C polymorphism 6,173 cases, 9,725 controls	MTHFR C677T, non-Hodgkin lymphoma	Association between both MTHFR C677T and A1298C and susceptibility to NHL; genetic models analyzed include allele contrast, heterozygote, homozygote, dominant, recessive	NS for pooled analysis
		MTHFR A1298C, non-Hodgkin lymphoma	Race/ethnicity	S: increased risk in Caucasians TT vs. CC; T vs. C
				S: decreased risk in Asians TT vs. CC; CT + TT vs. CC; T vs. C
			Tumor type	S: increased risk in Asians CC vs. AA; AC + CC vs. AA, C vs A
			Source of control	S: increased risk for follicular lymphoma TT vs. CC; TT vs. CT + CC
				NS
Wang [50]	19 studies 9,799 cases, 11,841 controls	SHMT C1420T, cancer risk	Association between SHMT C1420T and cancer using genetic models of heterozygote, homozygote, dominant, recessive.	NS
			Race/ethnicity	S: decreased risk in Asian population TT vs. CC; CT vs. CC; TT + CT vs. CC; TT vs. CT + CC
			Tumor type	S: decreased risk for colorectal cancer TT vs. CC; TT vs. CT + CC
			Source of control/population	NS
Wang [51]	8 case-control studies 3,232 cases 4,077 controls	SHMT C1420T, non-Hodgkin lymphoma	Association between C1420T and non-Hodgkin lymphoma using genetic models of allele contrast, heterozygote, homozygote, dominant, recessive. T vs. C	S: increased risk
			Ethnicity	NS
			Tumor type	NS

S = statistically significant (*P* < 0.05)

NS = not statistically significant (*P* > 0.05) or confidence interval includes 1

FA = folic acid

GI = gastrointestinal

GU = genitourinary

RCT = randomized control trial

CRC = colorectal cancer

MTHFR = methylenetetrahydrofolate reductase

### Supplementation

Several meta-analyses have evaluated the impact of folic acid (FA) supplementation on cancer risk using data from primary prevention trials for a number of conditions reporting cancer incidence. Two meta-analyses of Randomized Controlled Trials (RCTs) and a meta-analysis with 12 RCTs and 7 observational studies, evaluated the impact of FA supplementation, with or without other B vitamins, on cancer risk. Doses of FA ranged between 0.4 and 40 mg/day. Only one study used 5-methyltetrahydrofolate (5-MTHF), the metabolically active form of folate at a dose of 560 mcg/day. However, when that study was removed it did not change results. No significant effect of FA supplementation was found on total cancer risk in two of the analyses [32•, 33•]. One analysis reported a “borderline significant” 7% increase in overall cancer risk; however, the confidence interval (CI) included 1 [34•]. Two analyses also found no significant association between FA supplementation and risk of colorectal, prostate, lung, breast, or hematological cancers [32•, 33•]; however, one found a 24% increased risk of prostate cancer [34•]. One found no effect with less common types of cancer and cancers of unknown origin [33•]; and another found a 53% lower risk of melanoma [32•].

In subgroup analysis, Qin found a 10% increased risk of cancer in studies where > 60% of participants were on lipid-lowering drugs and hypothesized that statin treatment interacting with homocysteine metabolism in the presence of FA supplementation may impact cancer risk [32•]. Wien reported a 21% increased risk of cancer with doses between 0.4 and 1 mg/day, but not with doses higher than 1 mg/day and a 19% increased risk in smokers [34•].

Additional analyses evaluating risk in specific cancer types found a significant 3% decrease in risk of renal cancer with 100-mcg FA supplementation in a dose-response analysis [31]. When comparing highest with lowest intake from food fortification and supplements, two studies reported an inverse association and one study found no effect on risk of colorectal cancer [35–37].

### Serum Levels

Zhang and colleagues found elevated homocysteine levels and folate deficiency, as determined by serum folate level, to be associated with increased overall risk of cancer in a meta-analysis of 83 case-control studies [11]. Folate level was inversely associated with most cancer types except prostate, bladder, pancreatic, and breast. Protection was seen in studies from Europe, Asia, the Middle East, and Latin America, but not the USA or Australia. Zhao and colleagues found that the highest compared to lowest serum folate level was associated with decreased risk of esophageal cancer [20].

Higher or at least adequate levels may also be protective for cervical cancer. Increased risk of cervical cancer was observed with deficiency per serum folate in Asian, but not American studies. Serum levels used to categorize deficiency were different between studies, and some were higher than what is reported in the literature, which may have impacted results. These findings need to be confirmed using consistent cutoffs categorizing deficiency [28].

Two analyses found increased risk of prostate cancer when comparing highest with lowest serum folate levels. Both included five of the same nested case-control studies [17, 24]. One group included one additional case-control study and one retrospective case-control study [17]. Wang’s dose-response analysis reported a 4% increase in risk with each 5 nmol/L increase in serum folate [24]. After removing a retrospective study that was contributing to significant heterogeneity, Tio’s group found the association was still significant. The study removed was conducted in Jamaica, where risk was much higher with high levels, and the authors posed the question of whether or not folate may play a role in racial differences seen in prostate cancer risk [17, 24].

The studies in these analyses either compared tertile 3 to 1 or compared low level with high level. Low levels were between less than 4.82 nmol/L and less than 10.9 nmol/L and high between less than 10.3 and 58.2 nmol/L. Only one study found an association, but pooled results were consistent with that study. Serum folate was non-fasting, which could impact results and reflect recent intake. Some studies used a level barely considered adequate as the “high” category making it difficult to interpret these findings. The authors mentioned that there was a low incidence of cancers and could have impacted results [24]. Future studies should use serial measurements of RBC folate with clear cutoffs to help clarify this association [17].

Additional analyses found no association when comparing highest with lowest serum levels and risk of renal [31], pancreatic [22], breast [14], or colon cancers [29]. Chuang and colleagues found an inverse association for studies using radioimmunoassay, surfacing issues related to methods, including testing measurements, storage of samples and vitamin degradation, and variations in cohorts and study design [29].

### Cancer Types, Intake, and Risk

Folate intake is associated with a protective effect for some cancers, little to no effect for others, and a potential for increased risk with higher intake. When comparing the highest to lowest intake of folate, higher intake was associated with a nearly 50% decreased risk for squamous cell carcinoma of the head and neck (HNSCC) [18]; 35% reduced risk for oral cavity and pharyngeal (OPC) [19]; 41% reduced risk for all histological types of esophageal [21]; 34% reduction in pancreatic [21, 22]; and 16% reduction in bladder cancers [25]. The prospective analysis for pancreatic cancer was only significant when a study was removed that had lower levels of folate intake then the other studies. That study had a mean high intake of 246 mcg/day, an amount below the recommended intake, compared to 350–700 mcg/day in other studies [21]. Heavy alcohol use with low folate status was associated with 4 times the risk of OPC compared with low alcohol and high folate intake [19].

Dose-response analyses have shown that every 100 mcg/day increase in folate intake was associated with a 4.3% decrease in risk of HNSCC [18]; 12% decrease in risk of esophageal cancer in studies other than Asian and American studies [20]; and a 7% lower risk of pancreatic cancer [22].

There was no reduction in risk seen with lung cancer until one study with all females was removed due to heterogeneity. Then, a 10% reduction in risk was observed [23]. The authors attributed this to a higher percentage of smokers in the males [23]. Similarly, no protective effect comparing highest with lowest quantiles of folate intake for risk of ovarian cancer was found until a study was removed due to heterogeneity. Then, a 27% reduced risk was seen with higher intake [26]. It should be noted that highest intake in the removed study had a cutoff that could have included intake below or at the Dietary Reference Intake (DRI) [26].

Meta-analyses of studies for dietary folate and total folate intake found no association with risk of prostate cancer. Low intake in studies ranged from 123.8 mcg/day to 411 mcg/day (diet) and 551.9 mcg/day (total) and high intake reported as 235 mcg/day to 669 mcg/day (diet) and 934.1 mcg/day (total). In many studies, low and high diet intake are both below the recommended daily amount or the cutoffs for high with low overlap, making it difficult to discern a true cut point for risk if one does exist [17, 24].

Recent studies regarding folate intake have found little to no association between folate intake and breast cancer, with the exception of those comparing higher alcohol consumption with lower folate intake [14–16, 52]. Chen et al. and Zhang et al. demonstrated a non-linear dose-effect relationship between dietary folate intake level and breast cancer risk [14, 15]. For prospective studies, Chen et al. showed a U-shaped dose-effect relationship for dietary folate intake level and breast cancer risk [14]. There was a significantly decreased breast cancer risk with dietary folate intake between 153 and 400 mcg compared with those of < 153 mcg, but there was not a statistically significant reduced risk with dietary folate intake > 400 mcg compared to < 153 mcg [14]. For case-control studies, Chen et al. found that an increment of 100 mcg dietary folate per day showed a 9% reduction in breast cancer risk [14]. Zhang et al. found a potential J-shape correlation between folate intake and breast cancer risk; daily folate intake of 200–320 mcg was associated with lower breast cancer risk, but the risk increased significantly with a daily folate intake > 400 mcg [15]. Liu et al. found that a 220 mcg/day increment in dietary folate intake was not associated with breast cancer risk [16]. Studies varied in the incremental dose used to assess a dose-effect relationship.

Du and colleagues found a “marginal negative association” between total folate intake and risk of endometrial cancer [27]. An 11% reduction in risk was observed between higher intake and risk; however, the finding was not statistically significant. Interestingly, the association was not found for retrospective studies conducted outside of N. America (11 out of 14 studies). In the highest category of folate intake, which ranged from 205.8 to 987.7 mcg between studies, they reported a 5% elevated risk for every 100 mcg/day increase in folate intake, suggesting a threshold effect. They also suggested that type II or non-estrogen-dependent cancers are more likely to be related to p53 mutations, DNA damage, and changes to cell proliferation, yet the type of endometrial cancer not distinguished in most studies [27].

### Gene Polymorphisms

Gene variants associated with the folate metabolic pathway disturb nutrient bioavailability and contribute to a range of diseases. Methylenetetrahydrofolate reductase (MTHFR) directly affects DNA synthesis and methylation and has been associated with increased risk of certain cancers. In a meta-analysis of 134 case-control studies, the MTHFR C677T polymorphism was significantly associated with increased tumor risk [38]. In stratified analysis of the same study, increased risks of stomach and esophageal cancer were observed and increased risk in Asian ethnicity was observed [38]. Mutation of the 677 gene has also been associated with increased risk of lung, hepatocellular, breast, brain, and ovarian cancer in Asian populations and breast cancer in Turkish population [7, 12, 26, 39, 42, 44]. While no association was observed for MTHFR and oral cancer, subgroup analysis revealed a statistically significant increased risk in heavy versus non-heavy drinkers [46]. Two meta-analyses showed no association between MTHFR C677T allele and glioma, while an increased risk of brain tumor and meningioma was associated with the heterozygous genotype [41, 42]. MTHFR TT has been shown to be positively associated with risk of thyroid cancer [48] and increased risk of non-Hodgkin lymphoma in Caucasians [49].

Conversely, MTHFR 677TT may have a protective effect against colorectal cancer, in aggressive forms of prostate cancer, and prostate and non-Hodgkin lymphoma among Asians [30, 45, 49]. Similarly, a decreased risk of oral cancer has been associated with the MTHFR 677CT genotype in Asians [46]. High total and dietary folate intake was inversely associated with colorectal cancer for the wild-type allele, whereas MTHFRTT showed a protective effect for colorectal with high total folate intake compared to low total folate intake [30].

The presence of the MTHFR A1298C variant has been significantly associated with increased risk for cervical cancer, globally and among Asians [40]. Homozygosity for A1298C in Asians has been associated with an increased risk of myeloid leukemia and non-Hodgkin lymphoma [47, 49]. Conversely, a decreased risk of liver cancer has been observed with the homozygous variant MTHFR 1298 CC overall and in Asian populations [43, 44].

Homozygosity for SHMT C1420T has been associated with a protective effect against colorectal cancer and overall cancer risk for Asian ethnicity [50]. Another study found no association between colorectal cancer and SHMT; however, subgroup analysis showed a significant decreased cancer risk with low folate intake in the presence of SHMT1 variation. Folate intake level was not defined. A positive association was found between SHMT1 and risk for non-Hodgkin lymphoma [51].

## Conclusion

A number of factors need to be considered when evaluating and comparing studies, including limitations in the current literature. There is a great deal of heterogeneity in the populations studied, including geographical and ethnic differences, presence of polymorphisms, use of data from both pre- and post-fortification time periods, and inclusion of populations with and without fortification. Also, many studies relating to folate intake are observational and use a one-time food frequency questionnaire, which does not consider change in diet over time, and may not account for other lifestyle factors, including alcohol intake. Different methods and cutoffs are used to categorize highest compared to lowest intake and there is variability in serum levels, with a single serum level used to evaluate risk. Cutoffs overlap making it difficult to discern a true cut point if risk of one exists. The length of time for follow up varies between studies and may not be adequate to evaluate impact on risk. This makes it difficult to form a single conclusion regarding the impact of folate on cancer risk. However, there are populations who appear to be at risk due to either inadequate or excess intake.

Inadequate folate intake or deficiency, as measured by serum levels, may increase risk for cancer, including cancers of the head and neck [18], oral cavity and pharynx [19], esophagus [21], pancreatic [21, 22], bladder [25], and cervix. Low intake combined with high alcohol intake may also increase the risk of breast cancer. Gene polymorphisms may add to risk in certain ethnic groups. Clinicians should aim to optimize dietary folate intake and/or consider supplementing intake in individuals at risk for or with known deficiency, for example those with alcohol dependence or malabsorption. Prospective studies are needed to identify what level of folate intake is needed to correct deficiency based on gene status and evaluate whether or not nutrient remediation in high-risk populations can mitigate cancer risk.

In addition, more research is needed to help understand if the protective effects of folate are weaker in populations with a relatively higher intake level from mandatory fortification and/or supplementation. Population concerns regarding fortification and increased cancer risk are challenged in the current research. Fortification status in studies did not have any impact on risk with supplementation [32•, 33•]. Fortification practices vary and increasing use of organic and gluten-free flours, which may not be fortified, may change population intake over time. More prospective, post-fortification work needs to be done in some populations. For example, the association between supplemental intake, elevated serum levels, and increased risk of prostate cancer should be explored in the context of gene variants. Clinicians should weigh the pros and cons of supplementation in the presence of adequate intake with individuals based on all risk factors for disease.

Finally, it is important to note that dozens of enzymes affect the efficiency of folate-related metabolic pathways, and polymorphisms in these enzymes may alter substrate availability of folate and/or other nutrients as well as gene expression. Future research efforts should be aimed at understanding how polymorphisms affect nutrient status in ethnic-specific populations, how nutrient remediation affects enzyme function in the presence of polymorphisms, and how these may influence risk [53]. Also, it is important to remember that additional nutrients are involved in one-carbon metabolism. By studying folate intake or serum levels in isolation, we may be missing the critical role of synergy among nutrients. For the clinician, it is important to evaluate each case individually and consider overall diet pattern in the context of all internal and external aspects that impact disease risk.
